# The Aryl Hydrocarbon Receptor (AHR): A Novel Therapeutic Target for Pulmonary Diseases?

**DOI:** 10.3390/ijms23031516

**Published:** 2022-01-28

**Authors:** Binoy Shivanna, Chun Chu, Bhagavatula Moorthy

**Affiliations:** Division of Neonatology, Department of Pediatrics, Baylor College of Medicine, Houston, TX 77030, USA; shivanna@bcm.edu (B.S.); chunc@bcm.edu (C.C.)

**Keywords:** aryl hydrocarbon receptor, hyperoxia, acute lung injury, chronic obstructive pulmonary disease, and bronchopulmonary dysplasia

## Abstract

The aryl hydrocarbon receptor (AHR) is a cytoplasmic transcription factor that is well-known for regulating xenobiotic metabolism. Studies in knockout and transgenic mice indicate that the AHR plays a vital role in the development of liver and regulation of reproductive, cardiovascular, hematopoietic, and immune homeostasis. In this focused review on lung diseases associated with acute injury and alveolar development, we reviewed and summarized the current literature on the mechanistic role(s) and therapeutic potential of the AHR in acute lung injury, chronic obstructive pulmonary disease, and bronchopulmonary dysplasia (BPD). Pre-clinical studies indicate that endogenous AHR activation is necessary to protect neonatal and adult lungs against hyperoxia- and cigarette smoke-induced injury. Our goal is to provide insight into the high translational potential of the AHR in the meaningful management of infants and adults with these lung disorders that lack curative therapies.

## 1. Introduction

The Ah receptor (AHR) was discovered during studies aimed at understanding the metabolism of carcinogenic polycyclic aromatic hydrocarbons (PAHs), such as benzo[*a*]pyrene (BP) and 3-methylchlanthrene [1,2]. It was found that the aryl hydrocarbon hydroxylase, which later was found to be cytochrome P450 (CYP)1A1 and 1A2, was regulated by the *Ah* locus and was formally renamed the AHR [3]. More recently, the AHR has been known to be involved in chemical surveillances [4] and in different homeostatic pathways [5,6].

### 1.1. Regulation of the Aryl Hydrocarbon Receptor (AHR)

The *AHR* gene consists of 11 exons and is localized to chromosome 7p15 [7] in humans, and chromosome 12 A3 [8] in mice. In both species, the *AHR* gene’s promoter contains several transcription activation sites in the GC-rich region that lack TATA and CCAAT boxes [9,10]. The basal expression of the *AHR* is regulated by the zinc-finger transcription factors, such as specificity protein (Sp) 1 and Sp3, that have consensus binding sites in the GC-rich region of the *AHR* promoter [9,11]. Additional factors that regulate *AHR* expression include transforming growth factor (TGF)-β [12], nuclear factor erythroid 2–related factor 2 (NRF2) [13], β-catenin [14], and peroxisome proliferator-activated receptor α (PPAR-α) [15]. Interestingly, these factors regulate the *AHR* gene in a cell-specific manner. For example, TGF-β activation downregulates the *AHR* gene at the transcriptional level in human A549 lung carcinoma cells [16], whereas, in human HepG2 hepatocarcinoma cells, TGF-β activation increases *AHR* promoter activity [17]. In addition to these factors, epigenetic factors regulate the *AHR* gene expression. Histone deacetylase inhibitors increase, whereas histone acetylase inhibitors decrease, *AHR* promoter activity, indicating that histone acetylation is an important regulator of *AHR* expression [18]. Likewise, DNA hypermethylation down-regulates AHR expression [19].

### 1.2. Structure of the AHR 

**Figure 1:**
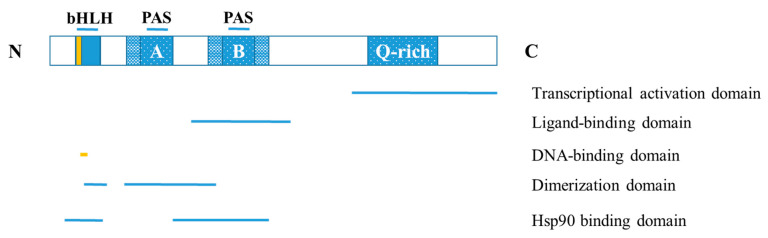
Structure of the AHR. bHLH: basic helix-loop-helix; PAS: PER-ARNT-SIM.

The human AHR protein has a molecular mass of 96 kDa and is composed of 848 amino acids, whereas the mouse AHR protein contains 805 amino acids and has a molecular mass of 90 kDa [20,21]. The AHR is a ligand-activated cytoplasmic transcription factor that belongs to the basic helix-loop-helix (bHLH) family [22]. The highly conserved b and HLH domains are located at the N-terminal of the AHR protein, where the former facilitates the binding of the transcription factor to DNA, and the latter promotes protein-protein interactions. Additionally, AHR contains two PAS domains, PAS-A and PAS-B, which have a homologous sequence to the protein domains found in the Drosophila genes period (Per) and single-minded (Sim) and the human AHR nuclear translocator (ARNT) [23]. The PAS-B domain contains the ligand-binding site [24]. The AHR protein’s C-terminal region contains the transactivation or Q-rich domain that participates in co-activator recruitment and transcriptional activation [25] (Figure 1).

### 1.3. The AHR Signaling Pathway

There are two pathways of AHR action: the classical pathway and the non-classical pathway.

#### 1.3.1. Classical (Canonical) Pathway

**Figure 2:**
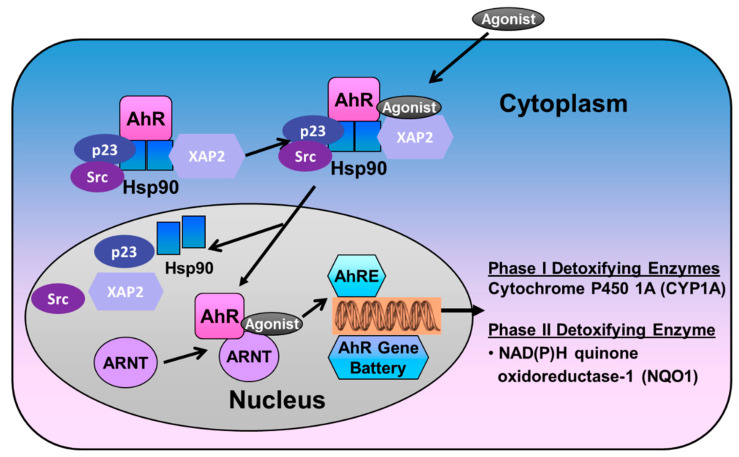
The AHR signaling pathway. Prior to ligand binding, the AHR (Aryl hydrocarbon receptor) is located in the cytoplasm as a complex comprising menu proteins, including Hsp90 (Heat shock protein 90), XAP2: (Hepatitis B virus X-associated protein 2), p23, and Src Kinase. Upon entry of the ligand-AHR into the nucleus, the associated proteins are dissociated, and the ligand-AHR complex binds to the ARNT (Aryl hydrocarbon receptor nuclear translocator,), which, in turn, binds to the AHRE (Aryl hydrocarbon receptor responsive elements) on the CYP1A1 promoter, leading to transcriptional activation of *CYP1A1* and other phase II genes.

The AHR is expressed practically in all mouse tissues [26], and, in humans, the receptor is highly expressed in the placenta, lungs, thymus, kidney, and liver [27]. The receptor is particularly enriched in lungs and placenta, tissues that participate in oxygen gas exchange [28]. The non-ligand bound AHR is predominantly cytosolic, localized in a core complex comprising two molecules of 90-kDa heat shock protein (Hsp90), the 23-kDa co-chaperone p23, and a single molecule of hepatitis X-associated protein-2 (XAP2), and the Src kinase [29,30]. The Hsp90 and p23 complex protects the receptor from proteolysis and facilitates ligand binding while preventing AHR from binding to the ARNT [31]. The XAP2 binds to the nuclear localization sequence (NLS) and prevents the translocation of non-ligand bound AHR to the nucleus [32]. Ligand-induced AHR activation results in a conformational change of the cytosolic AHR complex and release of XAP2 that exposes the NLS, resulting in translocation of this complex into the nucleus [33,34,35]. In the nucleus, Hsp90, p23, and Src kinase dissociate from the AHR, exposing the PAS domains, which facilitates AHR to dimerize with the ARNT [36]. The AHR/ARNT heterodimer complex then initiates transcription of many phase I (such as cytochrome P450 (*CYP)1A*) and phase II genes (anti-oxidant enzymes (AOE), such as glutathione S-transferase-α (*GST-α*), and NAD(P)H quinone reductase-1 (*NQO1*)), by binding to the xenobiotic responsive element (XRE)/AHR responsive elements (*AHRE*) motifs that contain the core bases 5′-*GCGTG-*3′ in the promoter region of these genes [37,38,39,40]. The AHR signaling is terminated upon the elimination of xenobiotics by at least two independent mechanisms, proteasomal degradation and competitive inhibition. AHR undergoes nuclear export, followed by E3 ubiquitin ligase-mediated ubiquitination and subsequent degradation by the 26S proteasome in the cytoplasm [41,42] (Figure 2). Recent evidence demonstrate that activation of autophagy can also degrade AHR protein via p23-dependent mechanisms [43]. In addition, the AHR signaling is terminated by a negative feedback loop via the AHR repressor (AHRR). Since it is structurally similar to AHR, the AHRR competes with the latter to dimerize with ARNT and bind to the XRE [44]. The XRE-bound AHRR recruits co-repressors, such as histone deacetylases, that repress transcription of the target genes [45].

#### 1.3.2. The Non-Classical (Non-Canonical) Pathway

Cross-talk between the AHR and other signaling mechanisms can result in non-canonical pathways of the action of AHR and its ligands [46]. In the nucleus, the AHR has been shown to associate with the hypophosphorylate form of pRB, resulting in growth arrest at the GL/S phase of the cell cycle [46,47]. Other mechanisms entail involvement of the transcription factor c-Maf [13,48], estrogen receptor, NRF2, RelA, and RelB [46].

### 1.4. AHR and Phase I/II Enzymes

The AHR’s well-established function is to mediate induction of phase I (CYP1A/1B1) and II enzymes that metabolize xenobiotics [49,50]. The phase I enzymes, such as CYP1A/1B1 monooxygenases and NADPH-CYP reductase, act to introduce reactive and polar groups to their xenobiotic substrates, which, in turn, leads to activation or detoxification of the substrates, leading to toxicity or excretion. These substrate modifications include hydroxylation, epoxidation, oxidation, reduction, hydrolysis, cyclization, and decyclization. In phase II reactions, enzymes, such as NQO1, glucuronyl transferases, and GST, conjugate the activated substrates with glutathione, sulfate, glycine, or glucuronic acid to detoxify the substrates and make them more polar so that they can be actively transported. Together, phase I and II enzymes detoxify toxic compounds and metabolites.

The CYP enzymes belong to a superfamily of hemeproteins that are involved in the metabolism of exogenous and endogenous chemicals [51]. The CYP1A enzymes are of particular interest to oxygen toxicity. The CYP1A subfamily has two isoforms, CYP1A1 and 1A2. CYP1A1 is essentially an extrahepatic enzyme that is predominantly present in rodent and human lungs, intestines, placenta, and kidneys. On the other hand, CYP1A2 is expressed mainly in the rodent and human liver and is not, or minimally, expressed in extrahepatic tissues. 

In addition, phase II enzymes, such as NQO1 and GST, have been shown to protect cells and tissues against oxidant injury induced by various toxic chemicals [52,53,54] and oxygen [55,56,57]. The protective mechanisms of these enzymes have been attributed to their ability to conjugate and excrete the reactive electrophiles and lipid peroxidation products generated by an oxidant injury [52,56].

### 1.5. Physiological Roles of the AHR 

The AHR is of particular interest to toxicologists, and extensive research has been conducted on its role in the bioactivation of polycyclic and aromatic hydrocarbons, leading to carcinogenesis [58]. Transgenic and knockout mice with AHR deficiencies have provided insight into the potential role(s) that AHR might play in normal physiological homeostasis [59,60]. The very fact that AHR is evolutionarily conserved from invertebrates who lack xenobiotic metabolism suggests that the role of AHR extends beyond xenobiotic metabolism. In fact, the AHR homolog spineless (ss) in Drosophila is necessary for the development of its legs, and distal segments of antennae [61] and AHR deficiency in *Caenorhabditis elegans* lead to defects in neuronal development [62]. Moreover, studies in knockout and transgenic mice indicate that AHR plays a vital role in the development of liver [63,64] and regulation of reproductive [65], cardiovascular [66,67], renal [68], hematopoietic [69], immune [70], and microbial [71] homeostasis. Additionally, AHR is known to regulate genes involved in proliferation, apoptosis, cell growth and differentiation, and cellular stress response [72].

## 2. AHR Ligands

Several structurally diverse compounds activate AHR. There are two types of AHR ligands, those coming from exogenous sources, such as diesel exhaust, commercial production, or industrial contamination (e.g., PAHs, PCBs, TCDD, etc.), or diet, or those generated endogenously (e.g., FICZ, indolo-carbazoles, indigoids, etc.) (Table 1).

### 2.1. Exogenous Ligands

The prototypical exogenous ligand is TCDD [73]. The majority of high affinity ligands are planar, hydrophobic halogenated hydrocarbons (HAHs) (e.g., TCDD, PCBs, dibenzofurans, biphenyls), and PAHs, such as MC, BP, benzanthracenes, benzoflavones, etc.) [73]. The most potent ligands are the ones that are most metabolically stable (e.g., HAHs), with binding affinities in the pM to nM range. The mechanisms of toxicity of HAHs involve the AHR, but PAHs in part mediate their action by inducing CYP1A1, which, in turn, bioactivates the PAHs to DNA-reactive metabolites, resulting in cancers of the lung and other extra-hepatic organs [73].

### 2.2. Endogenous Ligands

The majority of these compounds are proligands, which are transformed into ligands before they can bind and activate AHR [28]. The tryptophan derivative FICZ is one of the most potent AHR ligands and inducers of CYP1A1 [28].

Several developmental deficits and physiological impairments in AHR-deficient mice indicate the presence of several endogenous AHR ligands, including phytochemicals, microbial bioproducts, and metabolites of indole, tryptophan, heme, and arachidonic acid [99,100]. Additionally, several nonclassical synthetic compounds, such as omeprazole (OM), lansoprazole, thiabendazole, and primaquine, can activate AHR-dependent gene expression indirectly. Although these compounds are not AHR ligands by themselves, they are thought to activate AHR-dependent gene expression, either via metabolic conversion into a ligand or by their ability to affect a cellular pathway that results in AHR activation [101,102,103,104,105]. The prototypical ligands, such as TCDD and MC, are unsuitable for clinical use because of their well-known toxicities. Hence, identifying novel non-toxic AHR ligands, such as OM, is important for developing the AHR as a clinically relevant therapeutic target in oxidant injury- and inflammation-mediated lung disorders. OM, a benzimidazole derivative, is a proton pump inhibitor that inhibits gastric acid secretion both in humans [106] and in animals [107]. It has been widely used in the management of gastric acid disorders in humans [106]. Several in vitro studies suggest that OM activates AHR in human and rat hepatocytes [108,109,110,111], and the mechanistic role of AHR in the induction of CYP1A enzymes by OM in vitro has been extensively studied [112,113,114]. Furthermore, OM activates AHR and attenuates hyperoxic injury in adult mice in vivo [96] and adult human lung H441 cells in vitro [112], which indicates that OM can be used as an AHR agonist to understand AHR biology in hyperoxia-mediated lung disorders. Importantly, these ligands can exert different molecular and cellular responses within the same cell, tissue, or species [115]. The mechanisms of these ligand-specific effects are unclear at this time.

### 2.3. Selective AHR Modulators

A number of studies have recently showed AHR to be ligands that are selective modulators (sAHRMs) [115]. In addition to binding of 2,3,7,8-teyrachlorodibenzo*-p*-dioxin (TCDD) and PAHs, the AHR plays an important role in maintaining cellular homeostasis and in pathophysiology of many human diseases, and studies are emerging that the AHR is an important drug target [116]. The AHR binds structurally diverse chemicals, such as pharmaceuticals, phytochemicals, and many endogenous ligands. Thus, the AHR ligands are sAHRMs that display organ, tissue and cell-specific AHR agonist activities, and their functional diversity is very similar to steroid hormone and other nuclear receptors [116].

### 2.4. Current Barriers/Limitations to Developing AHR Ligands as Therapeutic Agents

The clinical applications of drugs using the AHR as a target have been lacking mainly due to the fact that the AHR was initially identified as the receptor that mediated the toxicity of (TCDD) and other polychlorinated aromatic environmental contaminants [117,118]. However, the discovery of many endogenous ligands, phytochemicals, and therapeutic compounds that activate the AHR suggest that AHR also plays a key role in myriad signaling pathways that regulate the normal physiology of the organism [85,117,119]. In fact, an AHR active drug, e.g., laquinimod, has been in clinical trials for treating multiple sclerosis [120].

## 3. Roles of the AHR in Lung Inflammation and Oxidative Stress

The recent discovery of the AHR as a crucial regulator of lung immune homeostasis suggests that AHR plays an important role in the modulation of lung inflammation. However, AHR biology in inflammatory lung disease is complex and is context- and disease-dependent. For example, depending on the nature of AHR ligands, the experimental conditions, and the disease model, AHR activation may potentiate or attenuate the lung inflammation [46,121]. Deficient AHR signaling has been reported to affect immune and non-immune cells, such as neutrophils, macrophages, and fibroblasts in the lung, leading to increased lung inflammation upon exposure to tobacco smoke, lipopolysaccharide, and hyperoxia [122,123,124]. Conversely, AHR activation has been shown to decrease airway inflammation in rodent models of asthma by regulating the production and secretion of Th2 cytokines, such as interleukin (IL)-4, IL-5, and IL-15 [125,126]. Interestingly, Wong et al. reported that AHR activation by TCCD increased the expression of the inflammatory cytokines, IL-1β, and monocyte chemoattractant protein-1 (MCP-1), in the mouse lungs, which they attributed to the increased lung infiltration of neutrophils and macrophages [126]. However, we observed that AHR activation by omeprazole (OM) decreased lung inflammation in an adult mouse model of acute hyperoxic lung injury, wherein both the neutrophils infiltration and MCP-1 expression were decreased compared to vehicle-treated animals [96]. These contrasting findings further emphasize the complexity of AHR biology in lung diseases, wherein the outcome is both ligand and context-dependent.

NRF2 is a master regulator of the antioxidant response, but it is also known to regulate the AHR expression transcriptionally. Moreover, the XRE and antioxidant responsive elements (ARE) are adjacent to each other in the promoter region of the genes encoding the antioxidant enzymes, such as NQO1 and GST [127]. Hence, AHR and NRF2 regulate and share a subset of common target genes with antioxidant properties, suggesting that AHR may be an essential regulator of the redox status of the cell. Along those lines, our studies in adult mice and adult human lung cells indicate that AHR deficiency increases, whereas AHR activation by OM decreases, oxidative stress in the lungs [96,112,128]. Additionally, AHR deficiency has shown to increase cardiac ROS levels via the pro-oxidant enzyme, NAD(P)H oxidase [129]. These observations strongly indicate that AHR signaling may be beneficial in inflammation- and oxidant injury-mediated lung disorders.

## 4. Lung Disorders and AHR

### 4.1. Acute Lung Injury

Acute respiratory distress syndrome (ARDS) is a life-threatening lung disease that is characterized by acute lung injury (ALI), respiratory failure, bilateral opacities on chest imaging, and a PaO_2_/FiO_2_ ratio < 300 mm Hg on at least a positive end-expiratory pressure (PEEP) of 5 or a PaO_2_/FiO_2_ ratio < 315 mm Hg without any PEEP requirement [130,131]. Despite improved intensive care management, the treatment of patients with ARDS is mostly supportive, with associated mortality as high as 46% [131]. The recent pandemic due to SARS-CoV-2 infection has, until today, seen numerous deaths (over 511,000) globally, and respiratory illnesses, such as pneumonia and ARDS [132], are the major causes of death. Thus, there is an urgent need for improved therapies for ARDS patients. Oxidative stress from increased reactive oxygen species (ROS) generation is a major contributor to ARDS development [133,134]. Supplemental oxygen, that is traditionally used as a life-saving measure in patients with impaired lung function, in itself, increases ROS generation and exacerbates lung injury [135,136,137]. Hyperoxia-induced acute lung injury in adult mice leads to a phenotype similar to human ARDS [138,139]. ALI is a multi-factorial morbid and fatal lung disorder in humans.

**Figure 3:**
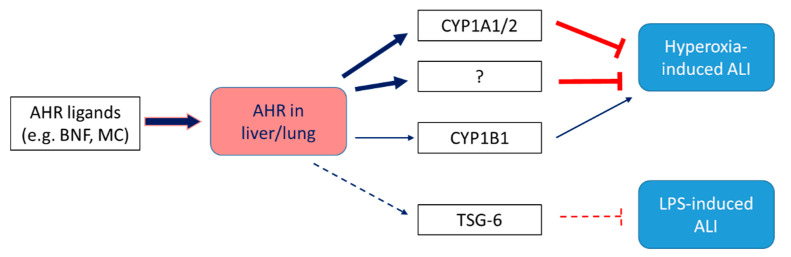
AHR modulates ALI in vivo via CYP1A enzymes. AHR is expressed in lungs and liver. In hyperoxic lung injury animal models, AHR deficiency potentiates the symptoms, which may be associated with AHR-regulated genes, such as CYP1A1/2, in these tissues. AHR-regulated genes may also alleviate LPS-induced lung injury. The CYP1A enzymes attenuate lung injury by detoxifying lipid hydroperoxides, such as F_2_-isoprostanes [129,152,153].

The AHR is expressed in numerous lung cells, including macrophages, club cells, alveolar type II cells, and endothelial cells [140,141,142,143,144,145,146], and plays a significant role in modulating lung function, especially in the context of environmental exposures-induced lung injury. In models of hyperoxic lung injury in adult animals, AHR deficiency potentiates hyperoxia-induced lung inflammation and damage [124,129], whereas AHR activation [124] mitigates these effects of hyperoxia. The molecular mechanisms by which the pulmonary AHR protects against hyperoxic lung injury remains poorly defined; however, CYP1A family of enzymes mediate some of the beneficial effects of the AHR in the context of hyperoxic injury. Hyperoxia for 48 h induces CYP1A1/1A2 in the liver and CYP1A1 in the lung of adult rodents. Interestingly, the induction of CYP1A enzymes in liver and lung decline after continuation of hyperoxia for 60 h [147,148], the time period that coincides with expression of overt respiratory distress in these animals, suggesting that CYP1A induction may protect against hyperoxic lung injury in adult rodents. The protection against hyperoxic lung injury of adult rodents pretreated with beta-naphthoflavone (BNF) [81] or 3-methylcholanthrene (3-MC) [149] has been attributed to the aryl hydrocarbon receptor (AHR)-mediated induction of CYP1A1, an enzyme with high peroxidase activity. It has also been shown that the CYP1A inhibitor 1-aminobenzotriazole potentiates hyperoxic lung injury in rats [124]. Studies have consistently demonstrated that CYP1A enzymes mitigate hyperoxic injury. Genetic or pharmacologic inhibition of CYP1A enzymes potentiates [124,150,151], whereas activation of these enzymes prevents and abrogate [81] hyperoxic injury. Mechanistic studies demonstrate that CYP1A enzymes protect against hyperoxic lung injury by decreasing lipid peroxidation and oxidative DNA damage [151,152]. On the other hand, CYP1B1, which is also regulated by the AHR, appears to play a pro-oxidant role in hyperoxic lung injury, as mice deficient in CYP1B1 are less susceptible to hyperoxic lung injury [152]. Recently, AHR activation was also shown to mitigate lipopolysaccharide (LPS)-mediated acute lung injury in mice by upregulating the immunomodulatory gene, TNF-stimulated gene 6 (TSG*-6*) [153] (Figure 3).

### 4.2. Chronic Obstructive Pulmonary Disease

Chronic obstructive pulmonary disease (COPD), a chronic adult lung disease that affects 300 million people worldwide, includes diseases such as chronic bronchitis and emphysema, and it is predicted by the World Health Organization to be the third most common cause of global deaths, by the end of 2030 [86,154]. The morbidity associated with the disease, including physician visits and hospitalizations, increases with age and is influenced by other comorbid diseases [155,156]. Further, COPD increases the economic and social burden and is predicted to be the seventh leading cause of disability-adjusted life years lost worldwide in 2030 [157]. COPD is a progressive lung disease that is characterized by mucociliary dysfunction and lung inflammation, fibrosis, destruction, and dysfunction and persistent airflow limitation [158]. Cigarette smoking is the most common risk factor for COPD [159]. Occupational exposures to organic and inorganic dusts, chemical agents and fumes, and indoor pollution from biomass cooking and heating are other important risk factors for COPD [154,160]. Additionally, genetics, lung developmental anomalies, and socioeconomic factors play important roles in the development and progression of COPD [159]. All the above mentioned risk factors ultimately cause oxidative stress, inflammation, and aberrant proliferation, death, and senescence of lung cells, leading to parenchymal tissue destruction and the development of COPD [161,162].

**Figure 4:**
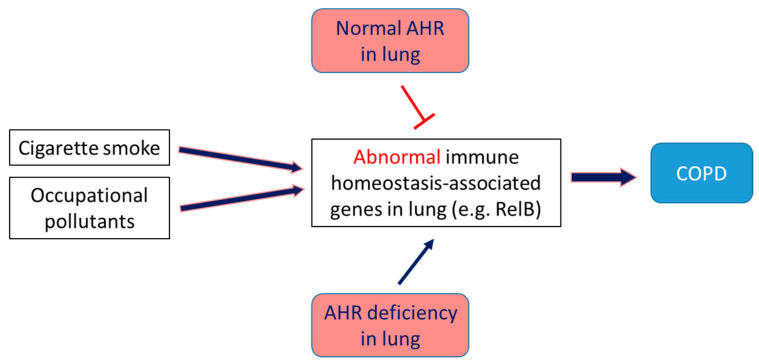
The role of AHR in the pathogenesis of COPD. Cigarette smoke and occupational pollutants may cause COPD due to abnormal immune homeostasis in the lung, which is attenuated by activation of AHR. It suggests AHR agonists may prevent or treat COPD.

The AHR exerts ligand-specific effects on the lungs and can either potentiate or attenuate COPD. For instance, the dioxins and PAHs in tobacco smoke and particulate matter mediate their toxic effects on the lungs through AHR signaling. These xenobiotic ligands induce inflammation, upregulate expression of mucin 5AC and matrix metalloproteinases, and damage ciliated cells, Club cells, and alveolar macrophages, contributing to the pathogenesis of COPD [75,126,163,164,165]. By inflammation and oxidative stress, the major contributors to the COPD pathogenesis [166]. Cigarette smoke (CS) exposure is a major risk factor for the development of COPD [167,168] and is a commonly used insult in animal models to elucidate the molecular mechanism of COPD [169]. Both acute and chronic CS exposure elicits an augmented neutrophilic response in the lungs of AHR-deficient mice than AHR-sufficient mice [122]. The precise molecular mechanisms through which endogenous AHR mediates these effects are unclear, but studies strongly indicate that the NF-κB protein RelB may be partly responsible. The AHR interacts with and modulates the expression of RelB [170,171], which is essential for maintaining immune homeostasis. AHR deficiency potentiates CS-induced RelB degradation, which, in turn, leads to: (1) increased expression of the neutrophil chemokine, intercellular adhesion molecule 1, and neutrophilia [123]; and (2) increased levels of the pro-inflammatory enzyme cyclooxygenase-2 via human antigen R-dependent pathway [75,121,122]. Further, AHR also regulates oxidative stress, the other common risk factor for COPD. AHR-deficient lung cells exhibit more increased reactive oxygen species (ROS) generation and decreased expression of the anti-oxidant enzymes, NQO1, and sulfiredoxin than AHR-sufficient cells, upon exposure to CS [172], suggesting that CS-induced oxidative stress is potentiated in AHR-deficient lungs. These findings collectively indicate that endogenous AHR ligands may protect the lungs against inflammatory and oxidant injuries and provide a mechanistic rationale for developing select AHR agonists as therapeutic agents to prevent and mitigate COPD (Figure 4).

### 4.3. Bronchopulmonary Dysplasia

Bronchopulmonary dysplasia (BPD) is a chronic lung disease of predominantly preterm infants that is characterized histopathologically by alveolar and pulmonary vascular hypoplasia [173,174,175]. The incidence of BPD remains unchanged despite significant advancement in the medical care of extremely low birth weight infants with respiratory dysfunction [176]. The therapies in the early phases of respiratory dysfunction in premature infants are mostly supportive, and there are no specific interventions known to prevent BPD directly. Furthermore, infants with BPD are more likely to have long-term pulmonary problems, increased re-hospitalizations during the first year of life, and neurodevelopment impairments [177,178,179,180,181,182,183,184]. In addition, BPD increases the economic burden with an estimated cost of BPD infants being twice that of non-BPD infants [185], making it the second most expensive childhood disease after asthma. Inflammatory stimuli, such as infection, hyperoxia, and mechanical ventilation, disrupt growth factor signaling, extracellular matrix assembly, and cell proliferation in the developing lungs and contribute to BPD pathogenesis [186,187,188,189]. Failure to understand the specific molecular mechanisms that contribute to the development of BPD is one of the main reasons for the lack of specific therapies to prevent BPD and its associated economic burden and long-term sequelae.

**Figure 5:**
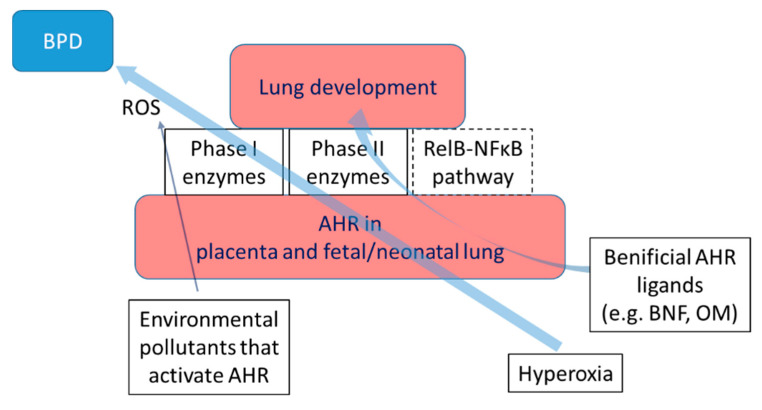
The role of the AHR in development of experimental BPD. Hyperoxia is one of major factors that contribute to the development of BPD. Some AHR ligands alleviate the hyperoxia-induced BPD, which may be associated with the activation of many AHR-regulated genes, such as phase I and II enzymes. However, other AHR ligands, such as environmental pollutants, potentiate the hyperoxia-induced BPD.

AHR signaling plays an important role in BPD pathogenesis [190,191]. In humans, placenta expresses the greatest levels of AHR followed by lungs and liver, whereas, in mice, the lungs express the highest levels of AHR followed by the placenta [28]. In human fetal lungs, the AHR is strongly expressed in the epithelial cells of the bronchus, bronchiole, and alveoli, and it is weakly expressed in the endothelial and smooth muscle cells of blood vessels [192]. Evidence indicates that AHR is expressed in the airway and parenchyma of the developing rodent lungs [97,144]; however, the lung cell-specific expression of AHR in rodents is not well characterized. Exposure to chronic hyperoxia activates AHR, as evidenced by increased expression of AHR-regulated phase I and II enzymes, such as CYP1A1 and NQO1, in wild-type (WT) mice but not in AHR dysfunctional (AHRd) mice. Interestingly, the failure of AHR activation in AHRd mice is associated with increased hyperoxia-induced lung inflammation and alveolar simplification. This implies that endogenous AHR signaling protects newborn mice against chronic hyperoxia-induced developmental lung injury [193]. By contrast, AHR activation protects neonatal rodents against hyperoxic lung injury. The AHR agonists, quercetin and β-napthoflavone, up-regulate the anti-oxidant enzymes, reduce oxidative adducts, decrease inflammation, and mitigate hyperoxia-induced neonatal lung injury in mice [82,83,194]. However, the AHR agonist, omeprazole, has differential effects on neonatal hyperoxic lung injury. While omeprazole activates AHR and mitigates hyperoxic lung injury in adult animals [96], prolonged (2-week) omeprazole therapy decreases pulmonary AHR activation and potentiates hyperoxia-induced: alveolar and pulmonary vascular simplification; inflammation; vascular injury; and oxidative stress [97]. In contrast, omeprazole activates AHR, increases surfactant and angiogenic proteins, and improves lung development and function in preterm rabbits exposed to hyperoxia [98]. Differences in the animal species, omeprazole dosage, and the nature and duration of the insult maybe some of the causes for these variable results. Nevertheless, these findings are consistent with the notion that an endogenous AHR response is protective in the context of neonatal hyperoxic lung injury. AHR activation can also potentiate neonatal lung injury in rodents. Maternal exposure to the environmental pollutant, BP, potentiates hyperoxia-induced alveolar hypoplasia in the offspring [195]. Mechanistic studies suggest that BP mediates hyperoxic injury by modulating the CYP1A/1B1 enzymes, leading to increased inflammation and oxidative lipid and DNA damage in the lungs [195]. Collectively, the findings indicate that AHR exerts ligand-specific effects on the developing lungs (Figure 5).

The AHR deficiency also potentiates hyperoxic injury in primary fetal human pulmonary microvascular endothelial cells (HPMECs), the cells which promote alveolarization and facilitate lung development. Silencing AHR signaling in primary fetal HPMEC increases hyperoxia-induced cytotoxicity, ROS generation, and inflammation and decreases the expression of antioxidant enzymes [146]. Interestingly, AHR-deficiency decreases the activation of the alternative NF-κB pathway (RelB) that mediates anti-inflammatory effects in these cells [146]. These results suggest that AHR signaling is also necessary to protect human fetal lung endothelial cells against hyperoxic injury. Gene expression profiling of AHR-sufficient and -deficient HPMEC exposed to hyperoxia indicate that AHR deficiency downregulates genes that mediate organ development and cell proliferation, and it upregulates genes that increase inflammation [145]. These results have important implications for managing BPD, a developmental lung disorder of preterm infants characterized by increased inflammation and interrupted alveolar development.

### 4.4. AHR Antagonists

Because the AHR is involved in the causation of the above mentioned lung diseases, one approach is to develop drugs and chemicals that target the AHR signaling pathway. The most well-known AHR antagonists are 3′methoxy-4′-nitroflavone (MNF) [94] and resveratrol [95], Recently, AHR activation has been shown to upregulate the expression of mucin SAC (oligomeric mucus/gel-forming (MUC5AC)) in the airway epithelial cell line via formation of ROS [196], which, in turn, contributes to lung diseases, such as COPD [197]. Chiba et al. [196] have shown that the AHR antagonist resveratrol mitigates the production of mucin. Wang et al. [74] have reported that the PAH BP increases dermiaogaphagoides group I (Der f1)-induced allergic lung inflammation via the AHR, and this effect is mitigated by the AHR antagonist CH223191. This AHR antagonist has also been shown to reverse the development of experimental pulmonary hypertension induced by Sugen 5146 in rats [198]. Development of AHR antagonists for human therapeutics is also being considered in the fields of wound healing and cancer [199].

## 5. Conclusions

The AHR is a versatile transcription factor that is evolutionarily conserved, serving many important physiological and pathological roles beyond its traditionally recognized role in xenobiotic metabolism. Importantly, activation of the AHR can exert opposing effects within the same cell or organ, depending upon the activating ligand and the nature of the insult. In general, endogenous AHR signaling is necessary to protect against both acute lung disease and chronic lung disorders, such as COPD and BPD. Furthermore, while the typical xenobiotic AHR ligands, such as TCDD and BP, can contribute to the development of lung diseases, the atypical AHR ligand, omeprazole, and the natural xenobiotic AHR ligands, quercetin and β-napthoflavone, can protect the lungs against oxidative damage. Despite decades of research, there are several knowledge gaps in the field of AHR biology. One of the most intriguing gaps is the mechanism behind the cell- and tissue-specific effects of the AHR ligands. The biological actions of the same AHR ligand can differ between tissues. There is also a lack of sufficient knowledge of the non-canonical pathways through which the AHR exerts its beneficial or harmful effects. Finally, the role of the negative feedback loop of the AHR pathway, e.g., AHRR, in the pathobiology is unclear. Deciphering these knowledge gaps would advance AHR biology and lay the foundation for selecting and developing the most effective AHR ligands as novel therapies for lung disorders, including ALI, COPD, and BPD.

## Figures and Tables

**Table 1:** List of major agonists (exogenous and endogenous ligands) and antagonists of the AHR. The table also describes the major target organs and the diseases that are modulated by the AHR.

Source	Examples	Target Organ/Disease
Exogenous	*Halogenated aromatic hydrocarbons*	Lung cancer [28,73]
	Dibenzofurans	Lung toxicity not confirmed
	Biphenyls	Lung toxicity not confirmed
	*Polycyclic aromatic hydrocarbons*	Lung cancer [28,73], asthma [74], COPD [75], chronic bronchitis [76,77]
	3-Methylcholanthrene	No severe lung toxicity
	Benzo[*a*]pyrene	Lung inflammation [78,79], respiratory tract cancer [80]
	Benzanthracenes	No immediate severe lung toxicity
	Benzoflavones	Non-toxic
Dietary Endogenous	*Flavonoids*	BPD/ARDS [81,82,83]
	Quercetin	BPD [84]
	Indole-3-carbinol	COPD, asthma, ARDS, BPD
	3,3′-Diindolylmethane	Lung cancer chemoprevention [85]
	Indolo[3,2-*b*]carbazole	No pulmonary therapeutic application reported
Tryptophan metabolites	Kynurenic acid	ALI [86]
	Kynurenine	Lung cancer [87]
	Tryptamine	No pulmonary therapeutic application reported
	6-Formylindolo[3,2-*b*]carbazole	LPS-induced ALI [88]
	Indoxyl sulfate	No immediate severe lung toxicity
Microbiota	3-Methylindole	May cause lung cancer [89]
	Tryptanthrin	Lung cancer [90]
	1,4-Dihydroxy-2-naphthoic acid	No pulmonary therapeutic application reported
	Indole-3-aldehyde	No immediate severe lung toxicity
	Indole-3-acetate	No pulmonary therapeutic application reported
	Phenazines	No pulmonary therapeutic application reported
	Indirubin	Lung cancer [91], anti-inflammatory [92]
	Malassezin	No pulmonary therapeutic application reported
Xenobiotic	3,4-Dimethoxy-*a*-naphthoflavone	Lung cancer [93]
	MNF	Lung cancer, COPD, asthma [94]
	CH-223191	Lung cancer, COPD, asthma [74]
Dietary	Resveratrol	Lung cancer, asthma COPD [95]
AHR Active Pharmaceuticals	Tranilast	COPD, Asthma [85]
	Leflunomide	BPD, ARDS [85]
	Omeprazole	BPD, ARDS [85,96,97,98]

## Abbreviations

AHR	aryl hydrocarbon receptor
AHRE	AHR responsive elements
AHRR	AHR repressor
ALI	acute lung injury
AOE	anti-oxidant enzyme
ARDS	acute respiratory distress syndrome
ARE	antioxidant responsive elements
ARNT	AHR nuclear translocator
bHLH	basic helix-loop-helix
BNF	beta-naphthoflavone
BP	benzo[*α*]pyrene
BPD	bronchopulmonary dysplasia
COPD	chronic obstructive pulmonary disease
CYP	cytochrome P450
GST-α	glutathione S-transferase-α
HPMEC	human pulmonary microvascular endothelial cells
Hsp	heat shock protein
MC	methylcholanthrene
MCP	monocyte chemoattractant protein
NLS	nuclear localization sequence
NQO1	NAD(P)H quinone reductase-1
NRF2	nuclear factor erythroid 2–related factor 2
OM	omeprazole
PPARα	peroxisome proliferator-activated receptor α
ROS	reactive oxygen species
TCDD	2,3,7,8-Tetrachlorodibenzo-*p*-dioxin
TGF	transforming growth factor
XAP2	hepatitis X-associated protein-2
XRE	xenobiotic responsive element
